# NF-κB Potentiates Caspase Independent Hydrogen Peroxide Induced Cell Death

**DOI:** 10.1371/journal.pone.0016815

**Published:** 2011-02-15

**Authors:** Jessica Q. Ho, Masataka Asagiri, Alexander Hoffmann, Gourisankar Ghosh

**Affiliations:** 1 Department of Chemistry and Biochemistry, University of California San Diego, La Jolla, California, United States of America; 2 Signaling Systems Laboratory, Department of Chemistry and Biochemistry, University of California San Diego, La Jolla, California, United States of America; Ohio State University, United States of America

## Abstract

**Background:**

The pro-survival activity of NF-κB in response to a variety of stimuli has been extensively characterized. Although there have been a few reports addressing the pro-cell death role of NF-κB, the precise mechanism of NF-κB's pro-cell death function still remains elusive.

**Methodology/Principal Findings:**

In the present study, we investigated the role of NF-κB in cell death induced by chronic insult with hydrogen peroxide (H_2_O_2_). Here, we show that NF-κB promotes H_2_O_2_ induced caspase independent but PARP dependent fibroblast cell death. The pro-death activity of NF-κB is due to the DNA binding activity of RelA, which is induced through IKK- mediated IκBα degradation. NF-κB dependent pro-survival genes, Bcl-2 and XIAP, were significantly repressed, while NF-κB dependent pro-death genes, TNFα and Fas Ligand, were induced in response to H_2_O_2_.

**Conclusions/Significance:**

We discovered an unexpected function of NF-κB, in that it potentiates chronic H_2_O_2_ exposure induced cell death, and suggest that NF-κB mediates cell death through the repression of pro-survival genes and induction of pro-death genes. Since unremitting exposure of tissues to H_2_O_2_ and other reactive oxygen species can lead to several degenerative disorders and diseases, our results have important implications for the use of NF-κB inhibitors in therapeutic drug design.

## Introduction

Mammalian cells are constantly exposed to reactive oxygen species (ROS), such as hydrogen peroxide (H_2_O_2_). Exogenous ROS arise from irradiation (UV, X-ray, γ-ray) and atmospheric pollutants, while endogenous ROS are mainly produced by the incomplete reduction of oxygen by cytochrome c during cellular respiration [1]. However, when the antioxidant capabilities of the cell are overwhelmed by ROS, a state of oxidative stress ensues, which can result in damage to DNA, proteins, and lipids [2]. Moreover, high and/or persistent levels of ROS result in aberrant cell death, which leads to aging and neurodegenerative disorders [3], [4]. In particular, ROS induced fibroblast cell death can cause chronic obstructive pulmonary disease [5], [6] as well as inadequate wound healing following myocardial infarction/reperfusion [7], [8]. ROS induces cell death by modulating cell signaling pathways. A prominent signaling pathway involved in mediating the cell survival/cell death fate is the nuclear factor-κB (NF-κB) pathway [3].

NF-κB is a family of transcription factors, which are comprised of five family members: RelA/p65, RelB, c-Rel, nfkb1/p50, and nfkb2/p52, that form homo- or hetero-dimers in a combinatorial manner. In resting cells, the NF-κB dimers are retained in the cytoplasm by forming stable complexes with NF-κB inhibitor molecules, IκB (α/β/ε). In the canonical activation pathway, stimulation with an extracellular stimulus, such as tumor necrosis factor α (TNFα), a pro-inflammatory cytokine, leads to phosphorylation of IκBα on serines 32 and 36 by IKK, the IκB kinase. This results in the ubiquitination of IκBα, which signals for the degradation of IκBα by the 26S proteasome. The freed NF-κB dimers can then translocate to the nucleus and activate transcription of their target genes [9], [10], [11].

Activation of NF-κB by exogenous H_2_O_2_ has been found to be highly cell type dependent, in which NF-κB is activated in a variety of cell lines such as Jurkat T cells and HeLa cells [12], [13], whereas NF-κB activation is inhibited in other cell lines such as murine neutrophils [14]. In cases where activation of NF-κB occurs, several mechanisms of NF-κB activation have been reported. While canonical activation of NF-κB via IKK-dependent IκBα degradation has been reported, other reports focus on an atypical mechanism of NF-κB activation in response to stimulation with H_2_O_2_ [15], [16]. This atypical mechanism involves an IKK independent mechanism and Tyr42 phosphorylation of IκBα, and only occurs in the absence of SHIP-1 [17], [18]. The pathway of NF-κB activation in other cell lines, such as in mouse embryonic fibroblasts (MEFs), has yet to be delineated.

The anti-cell death role of NF-κB has been extensively characterized. RelA deficient cultured cells undergo apoptotic cell death upon treatment with TNFα due to deficiencies in pro-survival and anti-oxidant gene transcription [19], [20]. RelA deficiency also leads to embryonic lethality accompanied by massive apoptosis in the embryonic liver [21]. In response to a variety of other stimuli, such as ionizing radiation and chemotherapeutic drugs, RelA also appears to have an anti-apoptotic effect [22]. Finally, NF-κB suppression of apoptosis in cancer cells is a central event in cancer biology, as well as in chemoresistance of tumor cells [23]. However, there have also been a few scattered reports addressing the pro-cell death function of NF-κB in response to atypical NF-κB activators [24], [25], [26], [27], [28]. Yet, the mechanism by which NF-κB mediates a pro-cell death response remains elusive.

In the present study, we sought to define NF-κB's role in immortalized MEF cell death induced by chronic insult with H_2_O_2_. Here we present evidence that unremitting exposure to H_2_O_2_ induces a caspase independent but PARP dependent cell death and that NF-κB potentiates cell death through the DNA binding activity of RelA, which is induced through the canonical activation pathway. Given that NF-κB dependent pro-survival genes, Bcl-2 and XIAP, were significantly repressed, while NF-κB dependent pro-death genes, TNFα and Fas Ligand, were induced in response to H_2_O_2_, we suggest that NF-κB mediates its pro- cell death function through the repression of pro-survival genes and induction of pro-death genes.

## Materials and Methods

### Cell culture, reagents, and antibodies

Immortalized MEFs cells were obtained from A. Hoffmann [29], cultured in humidified incubators at 37°C, 5% (v/v) C0_2_ and were grown in Dulbecco's modified Eagle's medium (DMEM) supplemented with 10% bovine calf serum (BCS) (Invitrogen) and 100 U/ml penicillin–streptomycin–glutamine (1xPSG) (Invitrogen). 293T were obtained from ATCC (cat # CRL-11268), and grown with DMEM supplemented with 10% Fetal Calf Serum (Invitrogen). Cells were stimulated for various periods with TNFα (Roche Biochemicals), glucose oxidase (Sigma), or H_2_O_2_ (Sigma). Cells were labeled with CFSE (5- (and 6-) carboxyfluorescein diacetate, N-succinimidyl ester) (Sigma), H_2_DCFDA (Invitrogen), or propidium iodide (Sigma). Cells were treated with z-VAD-fmk (caspase inhibitor VI) and DPQ (PARP Inhibitor III), which were both purchased from Calbiochem. Antibodies against IκBα (sc371), IKKα (sc7184), RelA(sc372), and αtubulin (sc5286) were purchased from Santa Cruz Biotechnology. Antibodies against IKKγ (#559675), pro-caspase 3 (#611048), active caspase 3 (#559565), PARP (#556362), and PAR (#551813) were purchased from BD Pharmingen. Bcl-2 antibody (#2876) was purchased from Cell Signaling and p53 antibody (#Op03) was purchased from EMD Biosciences.

### Generation of oxidative stress

Cells were first grown to 95-98% confluency. GO was dissolved in 50 mM sodium acetate pH 5.1, and added to fresh media supplemented with 10% BCS, except in the cases where RNA was extracted, in which serum free media was used.

### Measurement of ROS production

Measurement of intracellular ROS production was carried out as described in [30].The resulting supernatants were scanned with a Fluoromax-P instrument (J. Y. Horiba, Inc.) using a bandpass of 5 nm and ex 492 nm/em 526 nm.

### Cell Death Assay

MEF cell death was determined as the normalized value of propidium iodide (PI) incorporation = 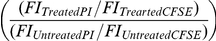, where FI =  fluorescence intensity of PI or CFSE of GO treated or untreated samples. A minimum of 2×10^6^ cells was used to label cells with 5 µM CFSE. Following GO treatment, each plate was then washed twice with 1×DPBS containing 100 mg/ml CaCl_2_ and Mg_2_Cl_2_, and treated with 10 µg/ml PI for 15 min in the dark. To remove adherent cells, cells were incubated with Puck's buffer (5.4 mM KCl, 0.14 M NaCl, 4.2 mM NaHCO_3_, 5.6 mM D-glucose dextrose, 10 mM Hepes, 1 mM EDTA, pH 7.4) for 15 min in the dark. For UV treated cells, floating cells were combined with the adherent cells. The fluorescence intensity for PI (ex 535 nm/em 617 nm) and CFSE (ex 492 nm/em 517 nm) were then taken with a Fluoromax-P instrument (J. Y. Horiba, Inc.) using a bandpass of 5 nm.

### Molecular Biology

IκBα constructs were cloned into the retrovirus vector pBabe-puro between the restriction sites *Eco*RI and *Sal*I. Mutagenesis reaction was performed with the Stratagene Quickchange Mutagenesis kit. The following primers were used to clone the *R35AY36A* Tg: forward: 5′- CGGGGCATGCGATTCGCCGCAAAATGCGAGGGGCGC-3′, reverse: 5′- GCGCCCCTCGCATTTTGCGGCGAATCGCATGCCCCG-3′.

### Retroviral transgenic system

293T cells were transfected using Lipofectamine 2000 (Invitrogen) containing 7 µg of the retroviral vector and 3 µg of pCl-Eco (Imgenex). Serum free DMEM containing the Lipofectamine and DNA mixture was removed 6 hrs later and replaced with DMEM supplemented with 10% FBS and 1xPSG. Cells were allowed to grow for 38–42 hrs post transfection. The media was then placed onto the *nfkb^−/−^* cells along with 8 µg/ml polybrene (Sigma). These cells were then grown for another 48 hrs before selection with 2.5 µg/ml puromycin (Calbiochem).

### Electrophoretic mobility shift assays (EMSAs)

EMSA experiments were carried out as described in [31] using a ^32^P- labeled oligonucleotide probe (5′-GCTACAA**GGGACTTTCC**GCTG**GGGACTTTCC**AGGGAGG -3′ **), which corresponds to the κB site of the HIV-1 LTR.**

### Western blots

Cells were lysed using RIPA buffer (20 mM Tris pH 7.5, 200 mM NaCl, 1% Triton-X 100, 2 mM DTT, 5 mM p- nitrophenyl phosphate, 2 mM sodium orthovanadate, 1X protease inhibitor cocktail [Sigma]). Membranes were developed using ECL chemiluminescence reagent (PerkinElmer) and quantitation of western blots was performed with ImageQuant TL (Amersham Biosciences).

### *In vitro* IKK kinase assay

Kinase assays were carried out as described in [29] using 2.0 µg of recombinant GST– IκBα (1–54). The reaction was visualized by PhosphorImager (Molecular Dynamics) and quantitated by ImageQuant TL.

### Annexin V staining

Cells were washed with PBS and resuspended in 100 µl of binding buffer (10 mM HEPES/NaOH, pH 7.4, 140 mM NaCl, 2.5 mM CaCl_2_). Alexa Fluor 647 conjugated Annexin V (BioLegend) and 7-AAD (BioLegend) were added according to the manufacturer's instructions, and the cells were incubated in the dark for 15 min at room temperature. The cells were analyzed using a Becton Dickinson FACSCalibur flow cytometer. Data analysis was performed with FlowJo software (Tree Star). Annexin V/7-AAD double-positive cells are necrotic and excluded from analysis.

### Quantitative Real Time PCR (qPCR)

RNA was isolated using the RNeasy kit (QIAGEN) and further purified with DNase I digestion (QIAGEN). cDNA was synthesized from 1 ug of RNA using the Superscript III First Strand synthesis kit (Invitrogen) and Oligo(dT) primers. Product accumulation was monitored by SYBR Green (Kapa Biosystems) fluorescence with Eppendorf Mastercycler ep *realplex*. The relative gene expression levels were calculated using the 2∧(-ΔΔCT) method [32], [33]. The p value was calculated using the Student's t test. The following primers were used: GAPDH Forward- AATGTGTCCGTCGTGGATCT Reverse- CATCGAAGGTGGAAGAGTGG, TNFα Forward- CCAGACCCTCACACTCAGATC Reverse- CACTTGGTGGTTTGCTACGAC, FasL (6753818a2) Forward- ACCCCCACTCAAGGTCCAT, Reverse- CGAAGTACAACCCAGTTTCGT, Fas Receptor (6679751a3) Forward- AATCGCCTATGGTTGTTGACC Reverse- TTGGTATGGTTTCACGACTGG, Bax (6680770a1) Forward- TGAAGACAGGGGCCTTTTTG Reverse- AATTCGCCGGAGACACTCG, XIAP Forward-GCAAGAGCTGGATTTTATGCTT, Reverse- TGCCCCTTCTCATCCAATAG, Bcl-2 Forward- ATGCCTTTGTGGAACTATATGGC, Reverse- GGTATGCACCCAGAGTGATGC, Bcl-xL (31981887a1) Forward- GACAAGGAGATGCAGGTATTGG Reverse- TCCCGTAGAGATCCACAAAAGT, cIAP-1 (6680696a2) Forward- TCAGTGACCTCGTTATAGGCTT Reverse- TCACACACGTCAAATGTTGGAA, p53 (6755881a1) Forward- GCGTAAACGCTTCGAGATGTT Reverse- TTTTTATGGCGGGAAGTAGACTG. All primers are provided in 5′ to 3′ fashion. Primers obtained from the Primer Bank [34], [35], [36] are given with the PrimerBank ID Accession number. GAPDH primers were obtained from [37], TNFα primers were obtained from [38], and Bcl-2 primers were obtained from [39]. XIAP primers were designed using Primer3.

## Results

### Continuous H_2_O_2_ exposure (via GO) to fibroblasts induces a caspase independent but PARP dependent cell death

**Figure 1:**
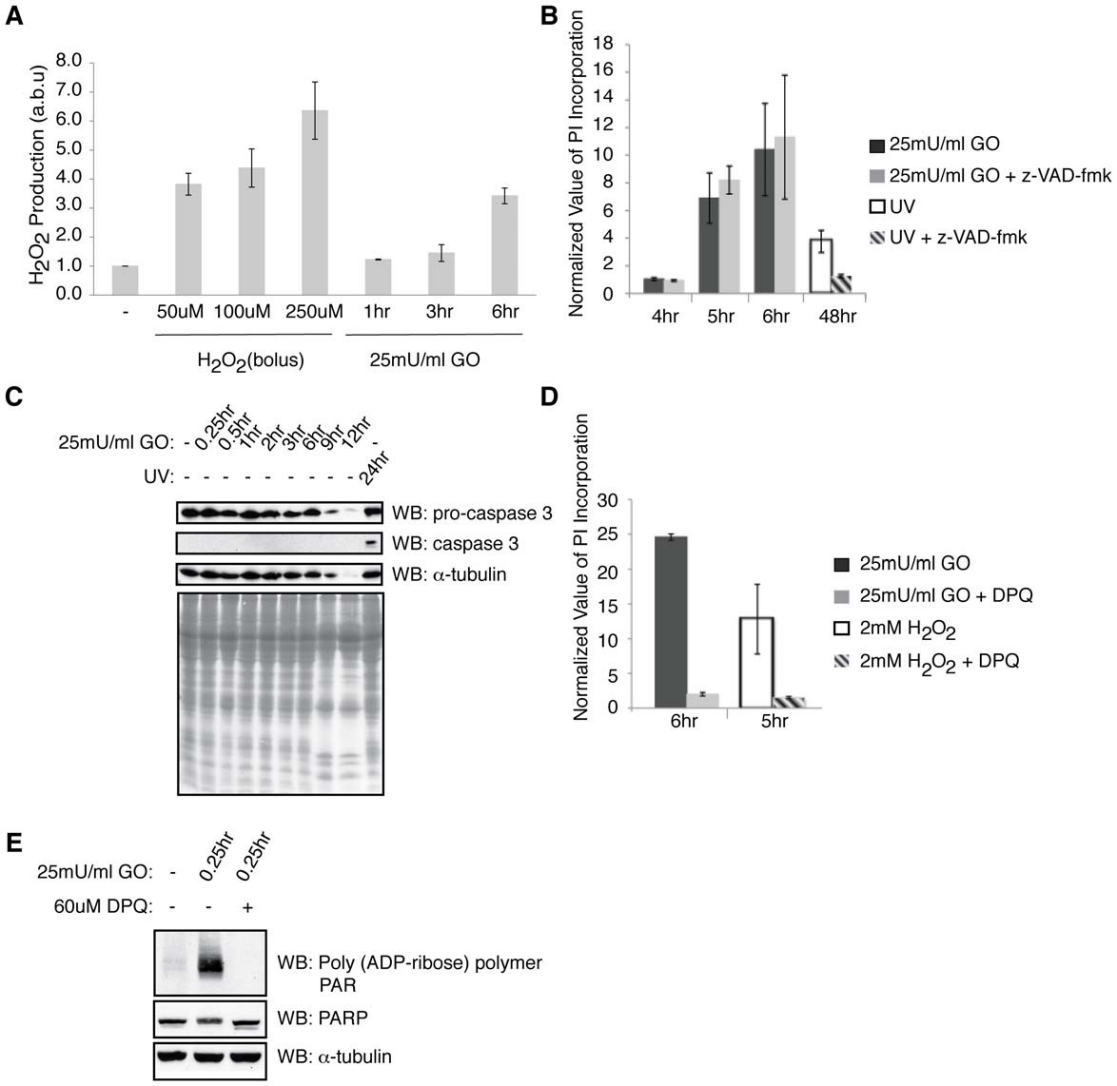
Continuous H_2_O_2_ exposure (via GO) to fibroblasts induces a caspase independent but PARP dependent cell death. (**A**) Wild type (wt) MEFs were either untreated (-) or treated with 25 mU/ml GO for the indicated periods of time. H_2_O_2_ readings for the H_2_O_2_ standard curve were taken 10 min after addition. All H_2_O_2_ levels are shown as normalized to untreated samples and are given in arbitrary units (a.b.u). (**B**) Following pretreatment for 1 hr in the presence of 100 µM z-VAD-fmk or DMSO, MEFs were subsequently treated with 25 mU/ml GO or 200 J/m^2^ UV and cell death was analyzed at the indicated time points. (**C**) Cell lysate was analyzed by western blotting against pro-caspase3, caspase3, and α-tubulin. The cell lysates were also stained by Coomassie Brilliant Blue to demonstrate equal loading. (**D**) Following pretreatment for 1 hr in the presence of 60 µM of DPQ or DMSO, MEFs were subsequently treated with 25 mU/ml GO or 2 mM H_2_O_2_ and cell death was analyzed at the indicated time points. (**E**) Cell lysate was analyzed by western blotting against PAR (poly (ADP-ribose) polymer), PARP, and α-tubulin. Both z-VAD-fmk and DPQ were kept in the media following GO or UV stimulation. Cell death was determined as the normalized value of propidium iodide incorporation (see Methods). All results are presented as the average of triplicate experiments. Error bars signify ±s.e.m (standard error of mean).

In chronic obstructive pulmonary disease and inadequate wound healing following myocardial infarction/reperfusion, chronic insult of ROS to fibroblast cells can lead to aberrant cell death [6], [7], [8]. We attempted to simulate this condition by generating persistent oxidative stress in immortalized MEFs with glucose oxidase (GO). It has been shown that addition of GO to the media produces continuous levels of intracellular H_2_O_2_, as opposed to a bolus addition of H_2_O_2_ [40], which we were also able to verify (J.Ho, data not shown). Intracellular ROS generation was verified by staining with DCFH-DA, a commonly used membrane permeable dye that fluoresces upon its oxidation to DCF by intracellular ROS [30]. Accordingly, ROS was produced in MEFs throughout the duration of treatment with 25 mU/ml GO (Fig. 1A). To roughly estimate the amount of intracellular H_2_O_2_ that GO was producing, known concentrations of H_2_O_2_ were also added in a bolus fashion to the cells and the intensity of fluorescence monitored. Based on the H_2_O_2_ standard curve, we estimated that, within the first 3 hrs of treatment with 25 mU/ml GO, less than 50 µM of H_2_O_2_ was being produced, and following 3 hrs, roughly 50–100 µM of H_2_O_2_ was produced (Fig. 1A).

We next determined the effect of prolonged H_2_O_2_ exposure (via 25 mU/ml GO) on MEFs. Significant cell death occurred within 5 hrs after GO treatment, as quantitated by the increase in propidium iodide (PI) incorporation, which correlates to a loss in membrane integrity. PI incorporation increased even further 6 hrs following treatment (Fig. 1B). Since an increase in oxidative stress can switch the cell death mode from a caspase dependent to a caspase independent cell death mode [41], we set out to determine whether GO generated H_2_O_2_ involved caspases. Treatment with irreversible general caspase inhibitor, z-VAD-fmk, was unable to prevent cell death. In agreement with these results, immunoblots against the cleaved form of caspase 3, one of the main executioners of apoptosis, revealed that cleaved caspase 3 is not present during treatment with GO (Fig. 1C). This is in contrast to UV induced MEF cell death, where caspases mediate cell death, as seen by the inhibition of cell death upon treatment with zVAD-fmk and by the production of the active form of caspase 3 (Fig. 1B, C).

Caspase independent MEF cell death induced by bolus addition of H_2_O_2_ occurs in a Poly(ADP-ribose) polymerase (PARP) dependent manner [42], which we were also able to confirm (Fig. 1D). PARP, a nuclear enzyme involved in the DNA damage response and cell death, becomes activated in response to DNA damage and attaches ADP-ribose units (PAR polymer) to itself and nuclear proteins [43]. We next sought to evaluate the contribution of PARP in cell death induced by continuous exposure to H_2_O_2_. Upon GO addition, cells pretreated with PARP inhibitor, DPQ, showed significant resistance to membrane permeabilization, as opposed to cells treated without DPQ (Fig. 1E). Given that PARP protein levels remained constant while PAR formation increased, as verified by western blot against PARP and PAR, we concluded that PARP was activated within 15 min of stimulation with GO. PAR formation was then eliminated upon pretreatment with DPQ (Fig. 1E). Thus, treatment of MEFs with GO results in a caspase independent but PARP dependent cell death.

### NF-κB potentiates H_2_O_2_ induced cell death

**Figure 2:**
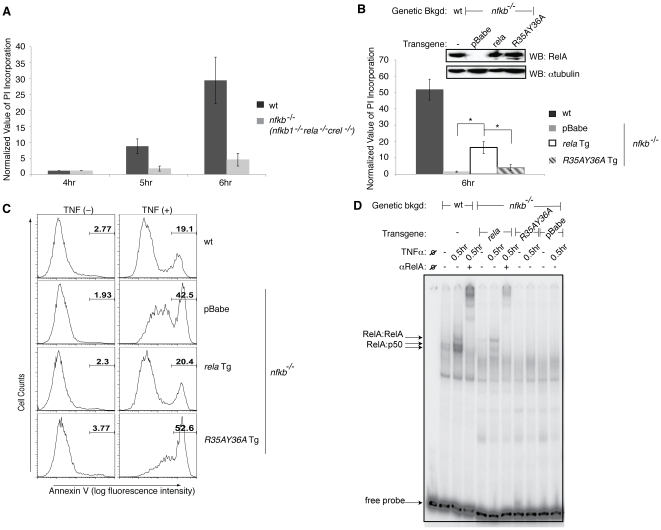
NF-κB augments cell death in H_2_O_2_ induced cell death. Cell death assays were performed on wt, *nfkb^−/−^* MEFs (**A**), and *nfkb^−/−^* cells reconstituted with empty vector (pBabe), *rela* transgene (Tg), or *rela* mutant (*R35AY36A* Tg) (**B**) treated with 25 mU/ml GO for the indicated periods of time. RelA was verified by western blot. Results are presented as the average of 3 independent experiments. Error bars signify ±s.e.m. * denotes p<0.05. (**C–D**) wt, *relA* Tg, *R35AY36A* Tg, and pBabe *nfkb^−/−^* cells were analyzed for cell viability in terms of AnnexinV positive cells following 16 hrs of treatment with 10 ng/ml TNFα (**C**) and for nuclear localization and DNA binding by EMSA analysis following treatment with 1 ng/ml TNFα (**D**). Supershift analysis was performed by adding RelA antibody to the EMSA reaction.

There have been extensive reports illustrating cooperativity between PARP and NF-κB. In response to DNA damaging agents such as ionizing radiation, PARP-1 has been shown to be an essential upstream mediator of NF-κB activation [44], [45]. PARP-1 can also act as a direct co-activator of NF-κB [46]. Since we have shown that chronic insult with H_2_O_2_ induces a PARP dependent cell death, we next wanted to determine whether NF-κB also plays any role in H_2_O_2_ induced cell death. To examine this, we compared rates of cell death in wt and *nfkb^−/−^* (*nfkb1^−/−^rela^−/−^crel^−/−^*) MEFs treated with 25 mU/ml GO. Although we expected that cell death would be enhanced in the absence of NF-κB due to its well established pro-survival activity, we found, to our surprise, that MEFs which lacked p50, RelA and c-Rel were more resistant to H_2_O_2_ induced cell death than wt cells (Fig. 2A).

To directly address the involvement of NF-κB in the delay of cell death, *nfkb^−/−^* cells were reconstituted, using a retroviral transgenic system, with either *rela* transgene (Tg), which is the major transactivating subunit of NF-κB, or a DNA defective binding mutant of *rela* (*R35AY36A* Tg). Both *rela* Tg and *R35AY36A* Tg reconstituted cells contained similar levels of RelA protein when compared to wt MEFs (Fig. 2B). Upon treatment with GO, *rela* Tg cells displayed increased incorporation of PI as opposed to *nfkb^−/−^* cells reconstituted with either empty vector (pBabe) or *R35AY36A* Tg (Fig. 2B). However the amount of PI incorporation in *rela* Tg cells was decreased compared to wt cells, indicating only partial rescue of the cell death phenotype. Our attempt to study cell death in *relA* Tg reconstituted in *rela^−/−^* cells was unsuccessful since reconstitution with *relA* did not rescue the cell death phenotype. This could be because of the transformation of the *rela^−/−^* cells with viral oncoproteins, which disrupts many transcriptional regulatory pathways, and resulted in the loss of this mechanism of modulating NF-κB function. Regardless, the lack of cell death in *R35AY36A* Tg in *nfkb^−/−^* cells indicates that NF-κB promoted cell death is dependent on NF-κB, in particular, RelA DNA binding activity. Additionally, we confirmed that the differences in the rate of cell death between wt and *nfkb^−/−^* cells reconstituted with either pBabe or *rela* Tg were primarily dependent on NF-κB. This was done by determining that all cell lines contained similar basal levels of p53, a well-known and potent contributor of cell death (Fig. S1).

Next, to examine the reason for the partial rescue of the cell death phenotype in response to GO, the response of the *rela* reconstituted cells to TNFα induced cell death, as well as TNFα induced DNA binding was inspected. Since it has been shown that *de novo* synthesis prevents the occurrence of apoptotic death upon treatment with TNFα [20], cells containing a transcriptionally active RelA should prevent cell death from occurring. Accordingly, upon TNFα stimulation, wt and *rela* Tg cells did not undergo cell death, as opposed to *R35AY36A* Tg and pBabe cells (Fig. 2C). EMSA results also show that NF-κB was not activated in both pBabe and *R35AY36A* Tg cells, in contrast to wt and, to a lower extent, *rela* Tg cells (Fig. 2D). The incomplete rescue of NF-κB activation in *rela* Tg cells might be due to the absence of NF-κB subunits, p50 and cRel. The p50:RelA heterodimer is the major NF-κB species, and both cRel and RelA homodimers have also been reported to play roles in the regulation of target gene expression [47]. Therefore, the absence of these dimers and the presence of only RelA homodimer alone might result in only suboptimal activation of some NF-κB target genes, and explains the partially functionally rescued *relA* reconstituted cells. Due to technical difficulties reconstituting p50 into *nfkb^−/−^* cells, we were unable to distinguish whether NF-κB's pro-cell death function is due to RelA homodimer or the p50:RelA heterodimer. However, from our data (Fig. 2B), it is clear that a RelA containing dimer is involved in promoting cell death.

### The canonical NF-κB activation pathway is required for the pro-cell death function of NF-κB in response to chronic exposure to H_2_O_2_

**Figure 3:**
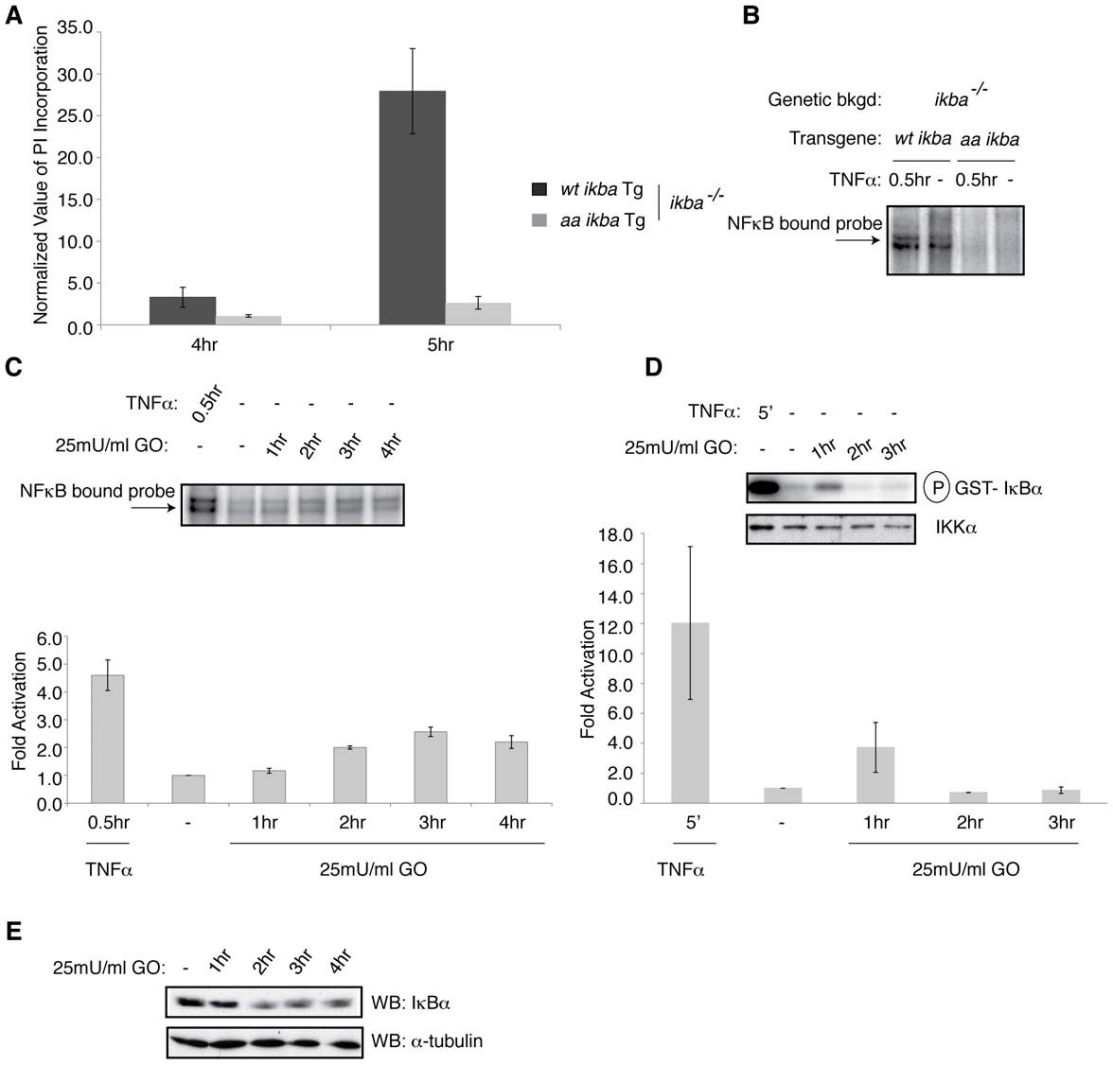
The canonical activation pathway is required for the pro-cell death function of NF-κB. (**A**) Cell viability assays were performed on *ikba^−/−^* MEFs reconstituted with either *wt ikba* Tg, or with *aa ikba* Tg treated with 50 mU/ml GO for the indicated periods. (**B**) *wt ikba* Tg and *aa ikba* Tg MEFs were treated with 1 ng/ml TNFα for 0.5 hr for EMSA analysis. (**C**) wt MEFS were treated with either 1.0 ng/ml TNFα or 25 mU/ml GO for the indicated times, for EMSA (**C**) IKK activity (**D**) or IκBα degradation analysis (**E**). Quantification of EMSA and IKK kinase assay experiments are shown. All results are presented as the average of 3 independent experiments. Error bars signify ±s.e.m.

To further determine whether NF-κB acts as a promoter of cell death in response to H_2_O_2_, we compared the rates of cell death of *ikba^−/−^* MEFs which were reconstituted with either wt IκBα (*wt ikba* Tg) or a mutant IκBα, where both Ser 32 and Ser36, which are the IKK phosphorylation sites, are mutated to alanine (*aa ikba* Tg), thus preventing NF-κB activation. Interestingly, in order to achieve cell death in a timely fashion, 50 mU/ml GO instead of 25 mU/ml GO, was needed to induce cell death in both cell lines. This may be a reflection of cell line-specific characteristics or due to the over-expression of IκBα upon reconstitution in these cell lines (G.Ghosh, data not shown). Nevertheless, *wt ikba* Tg cells had a rapid rate of cell death compared to *aa ikba* Tg cells, where the canonical NF-κB activation pathway had been blocked (Fig. 3A). As expected, NF-κB activation was completely blocked in *aa ikba* Tg cells as demonstrated by the lack of NF-κB activation upon TNFα stimulation by EMSA analysis (Fig. 3B). These results further suggest that the resistance to death of *aa ikba* Tg cells is due to the lack of NF-κB activity. All together, these results strongly indicate that NF-κB plays a pro-cell death role in response to H_2_O_2_ induced caspase independent cell death, and that the canonical activation pathway is required in mediating NF-κB's pro-cell death role.

We next examined whether NF-κB was activated in response to chronic exposure to H_2_O_2_. Indeed, as seen by EMSA analysis, NF-κB was activated in a prolonged manner upon addition of GO in MEFs (Fig. 3C). Given that NF-κB has been reported to be activated in both an IKK independent and dependent manner in response to H_2_O_2_, we then performed IKK activity assays, in which IKK was immunoprecipitated from GO treated MEFs followed by an *in vitro* kinase assay. IKK activity assays reveal that IKK is activated following one hour of treatment with GO (Fig. 3D). Accordingly, there is also concomitant degradation of IκBα (Fig. 3E). All together these results show that NF-κB is activated via the canonical pathway in MEFs in response to chronic exposure to H_2_O_2_.

### NF-κB-dependent survival genes are repressed whereas death promoting genes are induced by H_2_O_2_

**Figure 4:**
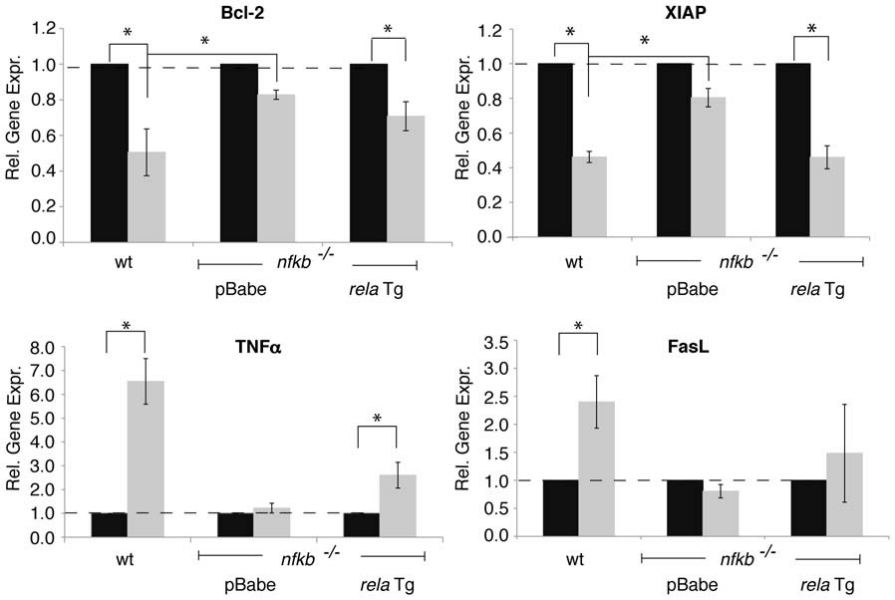
NF-κB dependent survival genes, Bcl-2 and XIAP, are repressed, while cell death genes, TNFα and FasL, are induced. Total RNA was isolated from wt and pBabe or *rela* reconstituted *nfkb^−/−^* MEFs without treatment (*black bars*) or following 4 hrs of treatment with 25 mU/ml GO (*grey bars*). Relative gene expression (Rel. Gene. Expr.) levels were determined using real time RT-PCR and are presented as compared to untreated samples. Results are presented as the average of three triplicate experiments. Error bars signify ± standard deviation. * denotes p<0.05.

As a transcription factor, NF-κB's active participation in cell death is likely to be mediated through its target genes. Therefore, we set out to examine the expression pattern of several NF-κB target genes that are known to impact cell death or survival (Table 1). Using real time quantitative PCR (qPCR), we measured mRNA levels at 0 and 4 hrs after GO treatment in wt MEF and *nfkb^−/−^* cells reconstituted with empty vector (pBabe) or *relA* Tg. While the majority of these genes did not undergo changes in expression levels (Table 1), we identified 4 genes that underwent significant alterations: cell survival factors, Bcl-2 and XIAP, and cell death promoting factors, TNFα and Fas ligand (FasL). The significant reduction of Bcl-2 gene levels in both wt and *rela* Tg cells and not pBabe cells (Fig. 4) implies that Bcl-2 repression is due to the presence of RelA. Decreased Bcl-2 protein levels were also observed only in wt, and not *nfkb^−/−^* cells, 6 hrs following treatment with GO (Fig. S2), suggesting that changes in mRNA levels correspond to changes in protein levels. A similar trend in gene expression is also seen for X-linked inhibitor of apoptosis protein, XIAP. In contrast, TNFα expression was significantly induced in both wt and *relA Tg* cells, while not in pBabe cells. FasL was induced in only wt cells. These results suggest that TNFα, but not FasL, induction by H_2_O_2_ is RelA dependent. The lack of FasL induction in *rela* Tg cells could be attributed to the partial rescue phenotype of the *relA* Tg cells (Fig. 2B).

**Table 1:** Relative gene expression levels.

Cell Survival Genes				Cell Death Genes			
	wt	pBabe	*rela* Tg		wt	pBabe	*rela* Tg
**Bcl-2**	0.51±0.13*	0.83±0.03*	0.71±0.08*	**TNFα**	6.5±1.00*	1.2±0.20	2.6±0.50*
**XIAP**	0.46±0.03*	0.80±0.05	0.50±0.07*	**Fas Ligand**	2.4±0.50*	0.8±0.12	1.5±0.90
**Bcl-xL**	0.79±0.12	0.91±0.11	0.67±0.20	**Fas Receptor**	1.1±0.07	0.8±0.04*	0.9±0.10
**cIAP-1**	0.89±0.24	0.87±0.03*	0.69±0.07*	**Bax**	0.9±0.27	0.9±0.11	0.76±0.08*
**p53**	1.01±0.11	0.84±0.08	0.90±0.15				

Note: Relative gene expression levels are presented as the average of three triplicate experiments with the ± standard deviation.

*denotes p<0.05.

## Discussion

This study has discovered an unexpected function of NF-κB in that it promotes MEF cell death in response to chronic insult with H_2_O_2_. We have shown that intracellular H_2_O_2_ was continuously produced in MEFs treated with GO and that this unremitting exposure to H_2_O_2_ resulted in a caspase-independent but PARP dependent cell death. We also show that the pro-death activity of NF-κB is dependent on the DNA binding activity of RelA, which is induced through IKK-mediated IκBα degradation. The death promoting activity of NF-κB might be mediated by the down regulation of a subset of NF-κB dependent pro-survival factors and up-regulation of NF-κB dependent pro-death factors, as demonstrated by the repression of Bcl-2 and XIAP, and induction of TNFα and FasL.

Due to the repression of pro-survival factor Bcl-2 in wt and *rela* Tg cells, we propose that the pro-cell death function of NF-κB is primarily due to its transcriptional down regulation of Bcl-2, since it has been shown that in MNNG induced caspase independent but PARP-1 dependent MEF cell death, over-expression of Bcl-2 can delay cell death [42]. Bcl-2 over-expression delays cell death due to its ability to prevent translocation of apoptosis inducing factor, AIF, from the mitochondria to the nucleus. This translocation event is central in causing MNNG and H_2_O_2_ induced MEF cell death [42]. Our proposed model is supported by two additional pieces of data. First, Bcl-2 protein levels decrease in wt MEFs as opposed to *nfkb^−/−^* MEFs after 6 hrs of treatment with 25 mU/ml of GO (Fig. S2), indicating that the changes in Bcl-2 mRNA level translates to changes in protein levels. Secondly, the fact that Bcl-2 over-expression can only delay cell death also fits with our proposed model, since *nfkb^−/−^* cells, which contain higher levels of Bcl-2 than either wt or *rela* Tg cells, eventually also succumb to cell death (J. Ho, data not shown). Additionally, our result, in which Bcl-2 is down regulated in a seemingly NF-κB dependent manner to promote PARP dependent fibroblast cell death, suggests a novel level of cooperativity between PARP and NF-κB. This is because PARP and NF-κB cooperativity has only been shown in instances where PARP-1 can act as a direct co-activator for RelA and p50, or as an upstream mediator of NF-κB activation in response to various stimuli [44], [45], [46].

We have convincingly demonstrated that NF-κB has a pro-cell death function in response to chronic insult with H_2_O_2_ in MEFs. Future experiments will be performed to determine whether this effect occurs in other cell types. Aside from MEFs, this type of caspase independent but PARP dependent cell death has only been reported to occur in cortical neurons, upon *N-Methyl-D-aspartic* acid (NMDA) over-stimulation [42], [48]. It is yet unknown whether NF-κB has any role in this NMDA induced, PARP dependent cell death in cortical neurons. Interestingly, it was shown that in HeLa cells, NF-κB promotes cell survival in response to a single bolus addition of low nanomolar concentration of H_2_O_2_ [13]. This variation can be attributed to differences in cell type, as well as differences in the application of H_2_O_2_, and thus amount of intracellular H_2_O_2_, which, as previously stated, can switch the cell death mode. We propose that NF-κB can be anti-cell death in caspase dependent cell death induced by transient or low levels of H_2_O_2_, but that NF-κB has a pro-cell death function in caspase independent cell death induced by continuous or high levels of H_2_O_2_. Indeed, NF-κB's role in promoting cell survival and death is a complex event. This is demonstrated by genotoxic agent, VP16, induced cell death, in which both pro- and anti-apoptotic genes were induced in NF-κB dependent manner, such that the final outcome depended on a balance of the induction levels of pro- and anti-apoptotic genes [39].

Our gene expression results are strongly supported by previous studies, which showed that cell death stimulation with daunorubicin, cisplatin, or p14^ARF^ over-expression in U2OS osteosarcoma cells resulted in RelA mediated transcriptional repression of pro-survival genes, Bcl-xL or XIAP [49]. These previous reports also showed that RelA acts in a dominantly transcriptionally repressive manner. Interestingly, we observe selective activation and repression of certain death and survival genes, in that TNFα was induced while Bcl-2 and XIAP were repressed in a seemingly NF-κB dependent manner (Fig. 4). There are currently more reports addressing NF-κB's singular transcriptional response in promoting cell death (either complete repression or induction of survival or death genes) [26], [27], [28] and significantly fewer reports describing NF-κB's mixed transcriptional response in promoting cell death (repression and induction of survival and death genes, respectively) [25]. This is the first report suggesting that NF-κB uses a mixture of transcriptional responses in promoting cell death upon ROS stimulation. It is unclear at this stage as to how NF-κB mediates repression of some target promoters and activation of others. However, given that oxidative stress is known to inactivate the cysteine active sites of cellular phosphatases, this would change the cellular phospho-protein state [18] and could potentially inhibit or enhance the recruitment of coactivators and corepressors. Additionally, RelA mediated repression of pro-survival genes has been reported to involve Thr505 phosphorylation of RelA, which can enhance the interaction between RelA and HDAC1 [49]. Further studies are still required to fully unravel this mechanism.

The induction and repression of cell death and survival genes, respectively, in response to H_2_O_2_ also suggests that activation of NF-κB is required for mediating its pro-cell death response. Previous reports of NF-κB's pro-cell death function have been shown to depend on either basal [24], [25] or activated NF-κB [49]. However, we cannot fully conclude that NF-κB activation is solely required to mediate the pro-cell death response, since pBabe reconstituted cells contain lower levels of basal NF-κB (Fig. 2D).

Our studies have also clearly shown that NF-κB is activated via the canonical pathway in MEFs, which is in contrast to reports where NF-κB activated in response to oxidative stress occurred via an atypical mechanism, involving an IKK independent mechanism and IκBα Tyr42 phosphorylation. The presence of SHIP1 has been reported to revert the mechanism of NF-κB activation from atypical to canonical in response to H_2_O_2_ [18]. Thus, the presence of SHIP2 in MEFs, which is functionally similar to SHIP1 [50], supports our observed NF-κB activation mechanism.

Unremitting exposure of tissues to ROS can lead to pathological conditions, such as neurodegenerative disorders and chronic obstructive pulmonary disease [3], [4], [6]. Our result that NF-κB might play a role in promoting cell death adds another layer of complexity to therapeutic drug design and should be taken into consideration when NF-κB inhibitor pharmaceutical targets are used in treatment. Overall, this study shows that NF-κB dependent transcription is responsible for promoting H_2_O_2_ induced cell death. Further experiments in future study will be done to explore the detailed molecular mechanism.

## Supporting Information

Figure S1 **wt and pBabe or** ***rela*** **Tg reconstituted** ***nfkb^**−**/**−**^*** **MEFs contain similar levels of basal p53.** Cell lysate of wt and *nfkb^−/−^* MEFs reconstituted with pBabe or *rela* Tg were analyzed by western blotting against p53 and α-tubulin.
(TIF)

Figure S2 **Bcl-2 protein levels significantly decrease in wt MEFs as opposed to** ***nfkb^**−**/**−**^*** **cells upon continuous exposure to H_2_O_2_.** wt MEFs and *nfkb^−/−^* MEFs were either untreated or treated with 25 mU/ml GO for 6 hrs. Cell lysate was analyzed by western blotting against Bcl-2 and α-tubulin.
(TIF)
