# Supplementary figures and images for: NF-κB Potentiates Caspase Independent Hydrogen Peroxide Induced Cell Death
Source: PLoS One. 2011 Feb 15;6(2):e16815. doi: 10.1371/journal.pone.0016815 (PMC3039651; doi:10.1371/journal.pone.0016815)

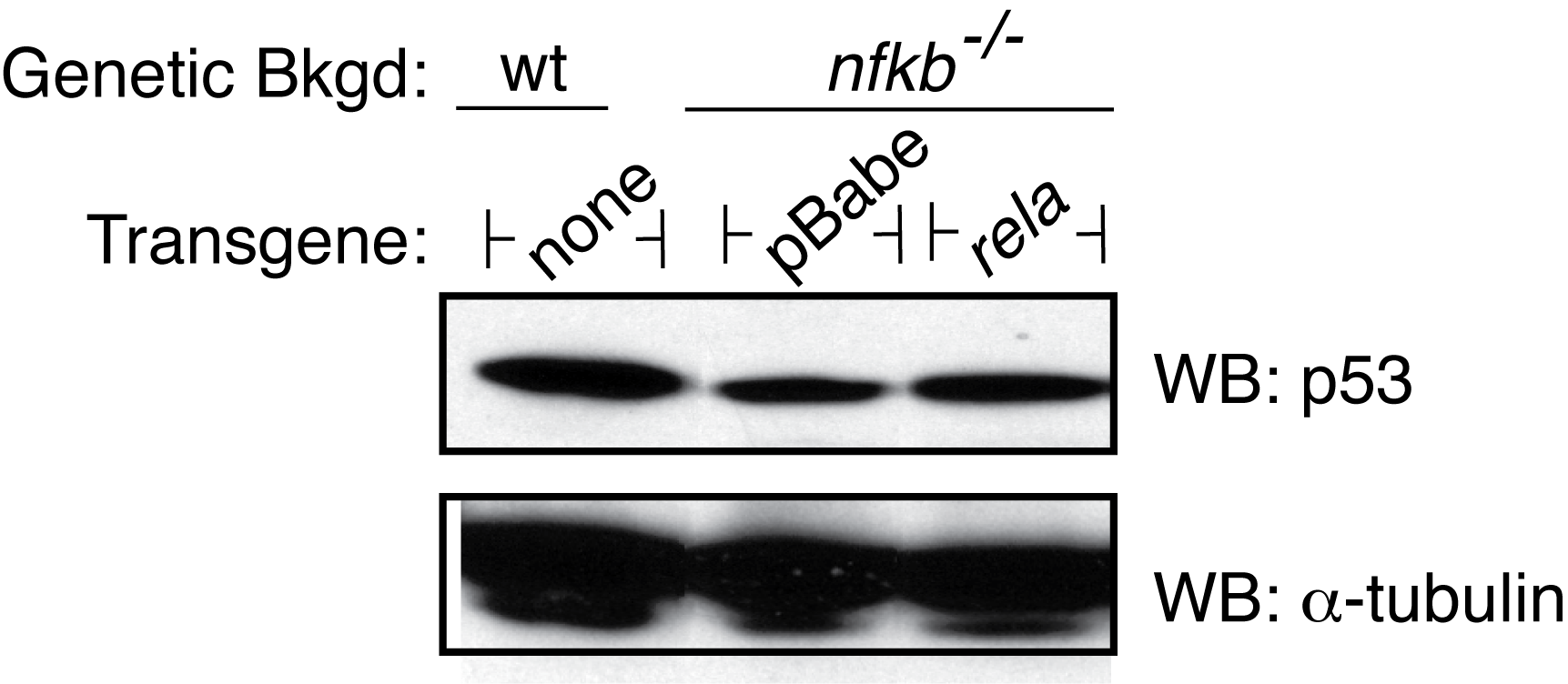

Supplement: Figure S1 — wt and pBabe or rela Tg reconstituted nfkb−/− MEFs contain similar levels of basal p53. Cell lysate of wt and nfkb−/− MEFs reconstituted with pBabe or rela Tg were analyzed by western blotting against p53 and α-tubulin. (TIF) [file pone.0016815.s001.tif]

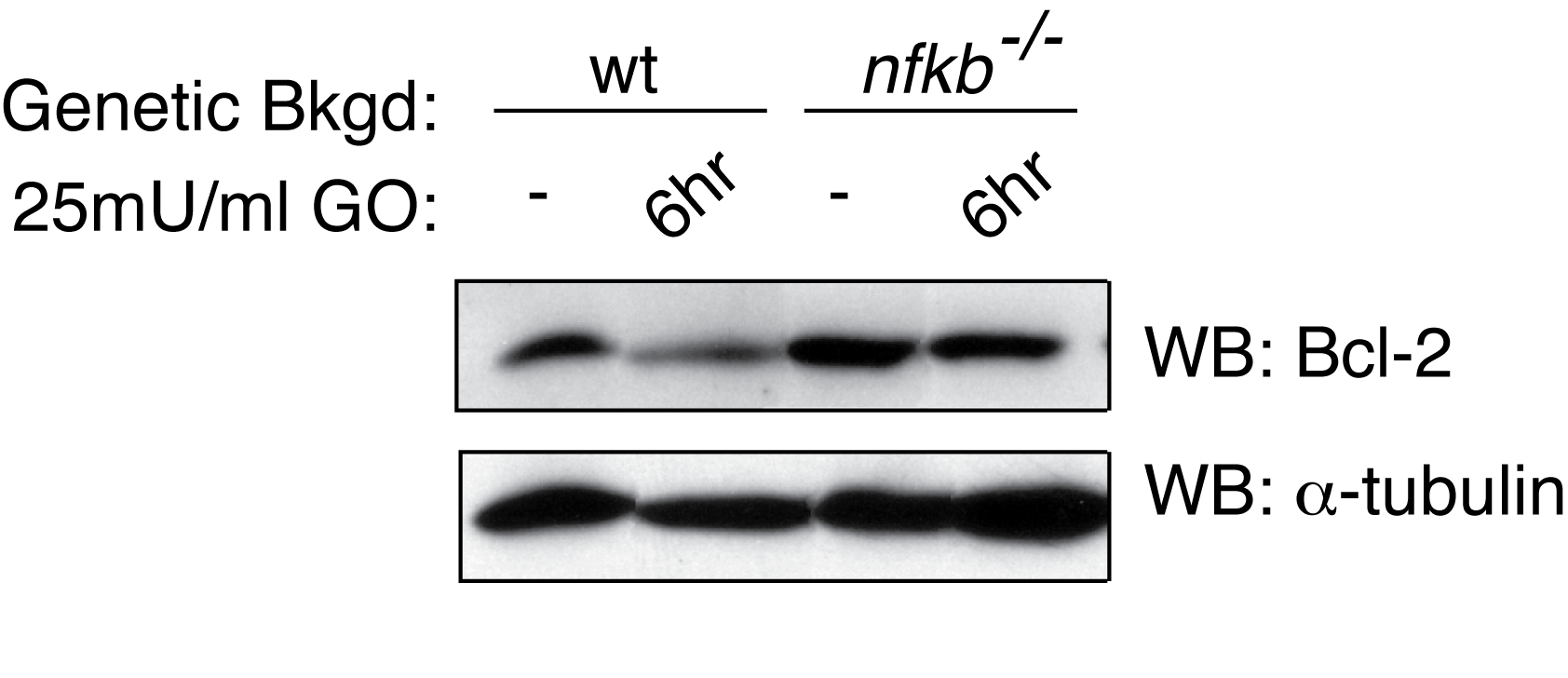

Supplement: Figure S2 — Bcl-2 protein levels significantly decrease in wt MEFs as opposed to nfkb−/− cells upon continuous exposure to H2O2. wt MEFs and nfkb−/− MEFs were either untreated or treated with 25 mU/ml GO for 6 hrs. Cell lysate was analyzed by western blotting against Bcl-2 and α-tubulin. (TIF) [file pone.0016815.s002.tif]
